# Skin sympathetic nerve activity in humans during exposure to emotionally-charged images: sex differences

**DOI:** 10.3389/fphys.2014.00111

**Published:** 2014-03-19

**Authors:** Rachael Brown, Vaughan G. Macefield

**Affiliations:** ^1^Integrative Physiology, School of Medicine, University of Western SydneySydney, NSW, Australia; ^2^Neuroscience Research AustraliaSydney, NSW, Australia

**Keywords:** skin sympathetic nerve activity, emotional processing, sex differences, sweat release, microneurography

## Abstract

While it is known that anxiety or emotional arousal affects skin sympathetic nerve activity (SSNA), the galvanic skin response (GSR) is the most widely used parameter to infer increases in SSNA during stress or emotional studies. We recently showed that SSNA provides a more sensitive measure of emotional state than effector-organ responses. The aim of the present study was to assess whether there are gender differences in the responses of SSNA and other physiological parameters such as blood pressure, heart rate, skin blood flow and sweat release, while subjects viewed neutral or emotionally-charged images from the International Affective Picture System (IAPS). Changes in SSNA were assessed using microneurography in 20 subjects (10 male and 10 female). Blocks of positively-charged (erotica) or negatively-charge images (mutilation) were presented in a quasi-random fashion, following a block of neutral images, with each block containing 15 images and lasting 2 min. Images of both erotica and mutilation caused significant increases in SSNA, with increases being greater for males viewing erotica and greater for females viewing mutilation. The increases in SSNA were often coupled with sweat release and cutaneous vasoconstriction; however, these markers were not significantly different than those produced by viewing neutral images and were not always consistent with the SSNA increases. We conclude that SSNA increases with both positively-charged and negatively-charged emotional images, yet sex differences are present.

## Introduction

Human emotion has long been studied, with numerous theories proposed and a diverse range of methods used to investigate emotional reactions and processing. One of the earliest theories of emotion based on empirical research is the James-Lange theory, which proposes that emotions are generated as a result of physiological events; a person feels sad *because* they are crying and not the other way around (James, 1884; Lange, 1885). However, the question of causation, as well as newfound knowledge on emotion processes, has meant that the theory has been largely abandoned (Golightly, 1953). There does remain a continuous development of emotional theories, although it is now clear that alterations in the activity of organs controlled by the autonomic nervous system (ANS) are involved in emotional state changes (Lacey and Lacey, 1970), such as when cutaneous flushing (vasodilatation) occurs in the face of a person who blushes when socially embarrassed.

The activity of the ANS and its broad range of physiological reactions are now widely studied during different emotional states or challenges, yet controversy still exists regarding the unambiguous outcome of these investigations (Hare et al., 1970; Callister et al., 1992; Lang et al., 1993; Fox, 2002; Ritz et al., 2005; Carter et al., 2008; Brown et al., 2012). There is a common perception that sex differences and emotion exist. Indeed, there is emerging evidence of sex differences in emotional processing, with females found to be more emotionally perceptive and experiencing emotions with greater frequency and intensity than males (Whittle et al., 2011), yet there is very little literature exploring sex and emotion. While it is known that there are profound sex differences in the prevalence of emotion dysregulation disorders (Gater et al., 1998), there are mixed results for those studies that have explored sex differences with respect to particular emotional processes (Bradley et al., 2001; McRae et al., 2008; Domes et al., 2010; Lithari et al., 2010; Bianchin and Angrilli, 2012).

Therefore, the aim of the present study was to expand on our previous study (Brown et al., 2012) in order to examine whether sex differences had an impact on the autonomic responses during presentation of neutral or emotionally-charged visual stimuli. By evoking emotional arousal passively we avoided the cognitive bias inherent in studies using mental stress, such as the Stroop color-word test or mental arithmetic. We wanted to use direct microneurographic recordings of skin sympathetic nerve activity (SSNA) and compare this to effector organ responses such as blood pressure, heart rate, respiration, and in particular sweat release and cutaneous blood flow, while showing subjects neutral or emotionally-charged images from the International Affective Picture System (IAPS)—a widely used set of visual stimuli (Lang et al., 1997). It is empirically obvious that emotional stimuli evoke sweat release and reduce skin blood flow (i.e., cold sweat), as well as cause the hairs to stand up (goosebumps); these effector-organ responses are produced by coactivation of cutaneous vasoconstrictor, sudomotor, and pilomotor neurons. While single-unit recordings of cutaneous vasoconstrictor and sudomotor neurons have been performed (Macefield and Wallin, 1996, 1999), though not during emotional stimuli, direct recordings of SSNA are typically multi-unit recordings—this offers the advantage that total sympathetic outflow to an area of skin can be measured. As sweat release is often utilized to infer increases in sympathetic outflow during studies on stress and emotion, and we know from our previous study that the correlation between SSNA and sweat release is poor, another aim of the study was to further cement the notion that direct recordings of SSNA provide a more robust measure of total sympathetic outflow to skin then sweat release alone.

## Methods

### General procedures

Studies were performed on 10 male and 10 female healthy subjects (age 20–46 years) Each subject gave informed written consent before participating in the study, and was told that they could withdraw from the experiment at any time, given that they were informed that they would be viewing some disturbing images. The studies were conducted under the approval of the Human Research Ethics Committee of the University of Western Sydney, and satisfied the Declaration of Helsinki. Subjects reclined comfortably in a chair in a semi-recumbent position with legs supported horizontally. Care was taken to ensure a calm and quiet environment to minimize spontaneous arousal responses. A comfortable ambient temperature was also maintained (22°C), as sympathetic outflow to the skin is susceptible to changes in ambient temperature. ECG (0.3–1.0 kHz) was recorded with Ag-AgCl surface electrodes on the chest, sampled at 2 kHz, and stored on computer with other physiological variables using a computer-based data acquisition and analysis system (PowerLab 16SP hardware and LabChart 7 software; ADInstruments, Sydney, Australia). Blood pressure was recorded continuously using finger-pulse plethysmography (Finometer Pro, Finapres Medical Systems, The Netherlands) and sampled at 400 Hz. Respiration (DC-100 Hz) was recorded using a strain-gauge transducer (Pneumotrace, UFI, Morro Bay CA, USA) wrapped around the chest. Changes in skin blood volume were monitored via a piezoelectric transducer applied to the pad of a finger; from this signal pulse amplitude was calculated using the Cyclic Measurements feature in the LabChart 7 software. A decrease in pulse amplitude was used to indicate a decrease in skin blood flow. Skin potential (0.1–10 Hz; BioAmp, ADInstruments, Sydney, Australia) was measured across the palm and dorsum of the hand; changes in skin potential reflect sweat release.

### Microneurography

The common peroneal nerve was located at the fibular head by palpation and superficial electrical stimulation through a surface probe (3–10 mA, 0.2 ms, 1 Hz) via an isolated constant-current source (Stimulus Isolator, ADInstruments, Sydney, Australia). An insulated tungsten microelectrode (FHC, Maine, USA) was inserted percutaneously into the nerve and manually advanced toward a cutaneous fascicle of the nerve while delivering weak electrical pulses (0.01–1 mA, 0.2 ms, 1 Hz). An uninsulated subdermal microelectrode served as the reference electrode and a surface Ag-AgCl electrode on the leg as the ground electrode. A cutaneous fascicle was defined as such if intraneural stimulation evoked paraesthesiae without muscle twitches at stimulation currents at or below 0.02 mA. Once a cutaneous fascicle had been entered, neural activity was amplified (gain 10^4^, bandpass 0.3–5.0 kHz) using a low-noise, electrically isolated, headstage (NeuroAmpEx, ADInstruments, Sydney, Australia). The identity of the fascicle was confirmed by activating low-threshold mechanoreceptors—stroking the skin in the fascicular innervation territory. The position of the microelectrode tip was then adjusted manually until spontaneous bursts of SSNA were identified. For identification purposes, individual bursts of SSNA were generated by asking the subject to take a brisk sniff or, with the subject's eyes closed, delivering an unexpected stimulus—such as a tap on the nose or a loud shout. Neural activity was acquired (10 kHz sampling), and sympathetic nerve activity was displayed as an RMS-processed (root mean square, moving average time-constant 200 ms) signal and analyzed on computer using LabChart 7 software. While direct sympathetic nerve traffic and cutaneous blood flow and sweat release were measured in different areas of the body, it is known that SSNA bursts generally appear in a bilaterally synchronous fashion in both the arm and leg nerves, and that there is a wide-spread activation of vasoconstrictor and sudomotor systems in response to arousal stimuli (Bini et al., 1980).

### Emotional stimuli

Emotional state changes were produced by viewing standard images from the International Affective Picture System (IAPS: Lang et al., 1997). Each picture used in the system has been extensively tested and rated for valency (its subjective impact, ranging from extremely negative to extremely positive) and arousal. In our study, positive emotions were evoked by viewing images of erotica with high positive valence ratings, while negative emotions were evoked by viewing images of mutilation with high negative valence; both sets had high arousal ratings. Once a suitable intraneural site with spontaneous SSNA was found and the subject was relaxed, a 2-min resting period was recorded, following which the subject was shown 30 neutral images, each image lasting 8 s, for a total of 4 min. This was followed by a block of 15 images (either erotica or mutilation) lasting 2-min. Images of erotica or mutilation were presented in a quasi-random fashion at a time unknown to the subjects, with each 2-min block of emotionally-charged images following a 2-min block of neutral images. In total, each subject viewed 3 blocks of erotica and 3 blocks of mutilation with 6 intervening blocks of neutral images. All subjects were naïve to the IAPS images.

### Analysis

Peak amplitudes of SSNA, measured over consecutive 1-s epochs, coupled with the total number of sympathetic bursts, were measured over each 2-min block. Visual inspection, coupled with auditory recognition of the neural signal, was used to identify individual bursts of SSNA. In addition, baseline was defined manually in the RMS-processed signal and the computer calculated the maximum amplitude above baseline. A beat-beat analysis was conducted for heart rate, blood pressure, skin blood flow, skin potential, and respiratory rate over each 2-min block and a mean value for each block in each subject was derived. A mean group value for each 2-min block could then be calculated and absolute changes derived. Absolute changes in skin potential and skin blood flow were normalized to the individuals average resting value. In addition to absolute changes for each 2-min block, relative changes normalized to neutral were calculated for the resting period and positive and negative images—the average of each neutral block was classified as 100% so therefore, values for the other blocks of images were expressed relative to that value. Analyses were conducted on pooled data, as well as after dividing the data into male and female groups. Repeated Measures Analysis of Variance of each physiological parameter across the three stimulus conditions, coupled with a Newman-Keuls test for multiple comparisons, was used for statistical analysis of the data (Prism 5 for Mac, GraphPad Software Inc, USA). In addition, paired *t*-tests were used to compare relative changes (normalized to neutral) in various physiological parameters for the erotica and mutilation data sets, and for the male and female groups. The level of statistical significance was set at *p* < 0.05.

## Results

**Figure 1:**
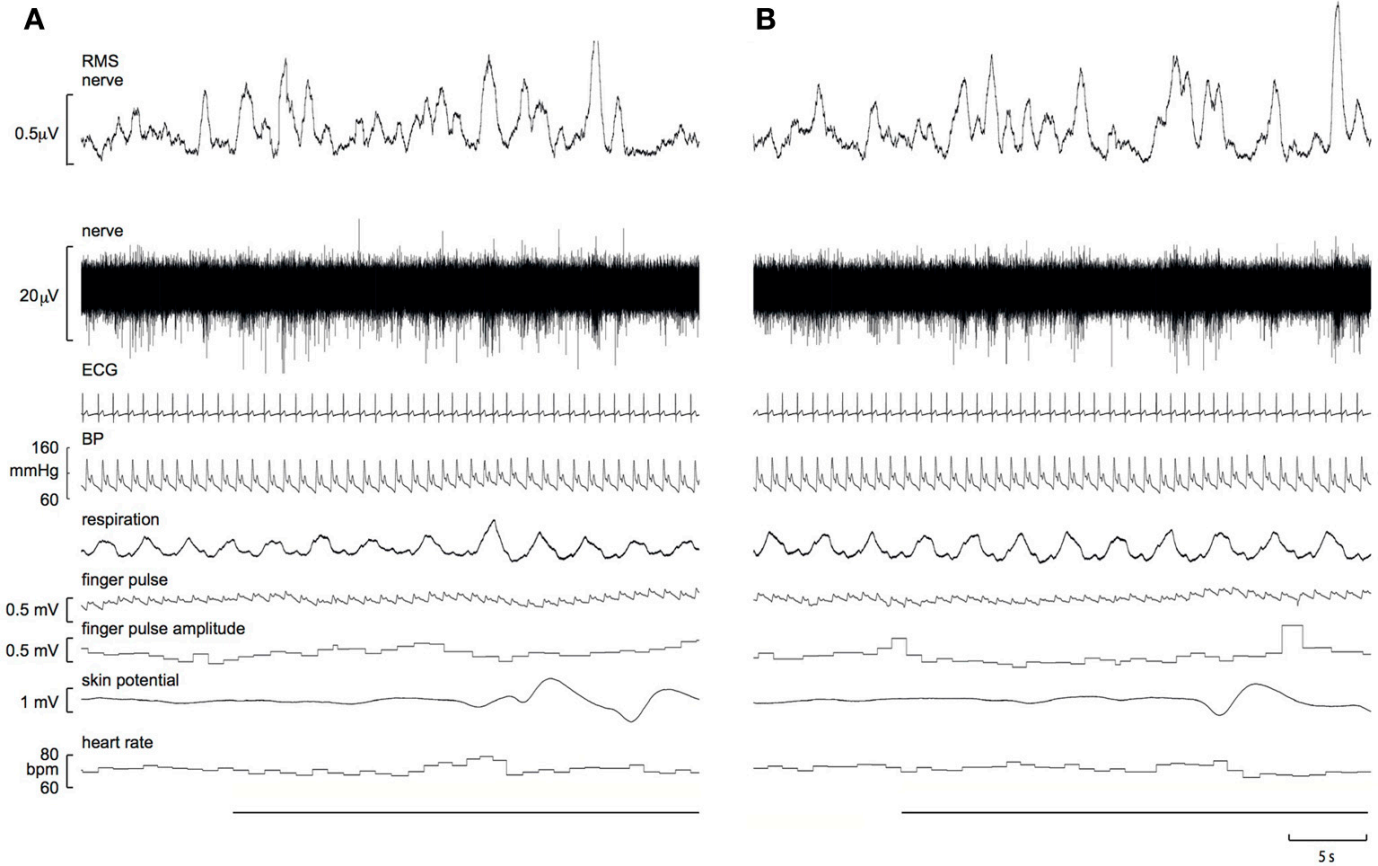
**Experimental records of skin sympathetic nerve activity, presented as the raw signal (nerve) and the RMS-processed version (RMS nerve), obtained from a 21 year-old male subject while viewing images of mutilation (A) or erotica (B)**. Note that the sympathetic responses are greater to erotica. A 10 s representative period of the preceding neutral images is presented at the start of each block. The horizontal black bars represent viewing of the emotionally-charged images.

Experimental records from a 21 year-old male, viewing images of erotica and mutilation, are shown in Figure 1. It can be seen that SSNA clearly increased during both stimuli, though the response to erotica was greater.

**Figure 2:**
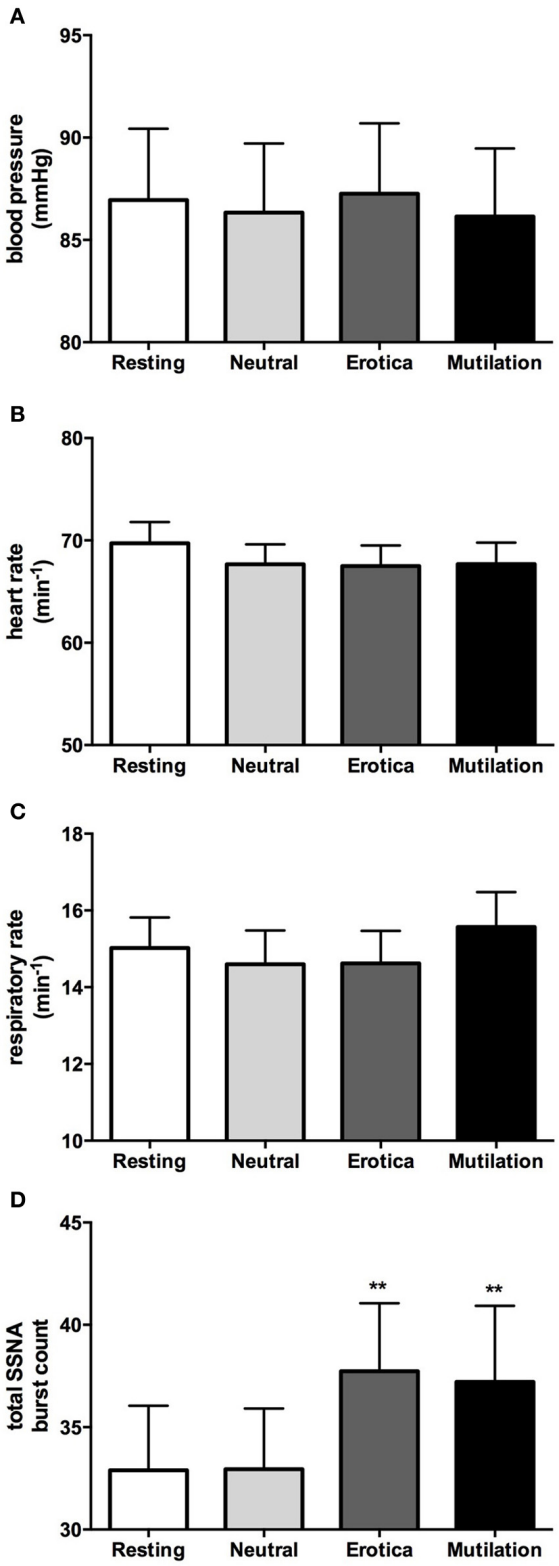
**Mean ± SE absolute values of blood pressure (A), heart rate (B), respiratory rate (C), and total burst count of skin sympathetic nerve activity (D) across the four conditions**. As can be seen, no statistical differences exist except for the SSNA burst count. Erotica and mutilation were statistically different from both resting and neutral. ^**^p < 0.01.

In accordance with our previous study (4), when males and females were grouped together, absolute values for blood pressure, heart rate, respiration, cutaneous blood flow, and sweat release showed no significant changes during viewing of emotionally-charged images, compared to viewing neutral images or at rest. SSNA however, did show significant increases when viewing either images of erotica or mutilation compared to the resting and neutral phases, although this was for burst frequency only (*p* < 0.05), not burst amplitude. Absolute values for blood pressure, heart rate, respiratory rate and total SSNA burst count at rest (no images), when viewing neutral images and when viewing images of erotica or mutilation, are illustrated in Figure 2. Likewise, relative changes normalized to neutral showed similar results to our previous study, with the only significant differences being seen in SSNA burst amplitude (erotica *p* = 0.044; disgust *p* = 0.028) and frequency (erotica *p* < 0.0001; disgust *p* = 0.002) during viewing of both positive and negatively-charged images.

**Figure 3:**
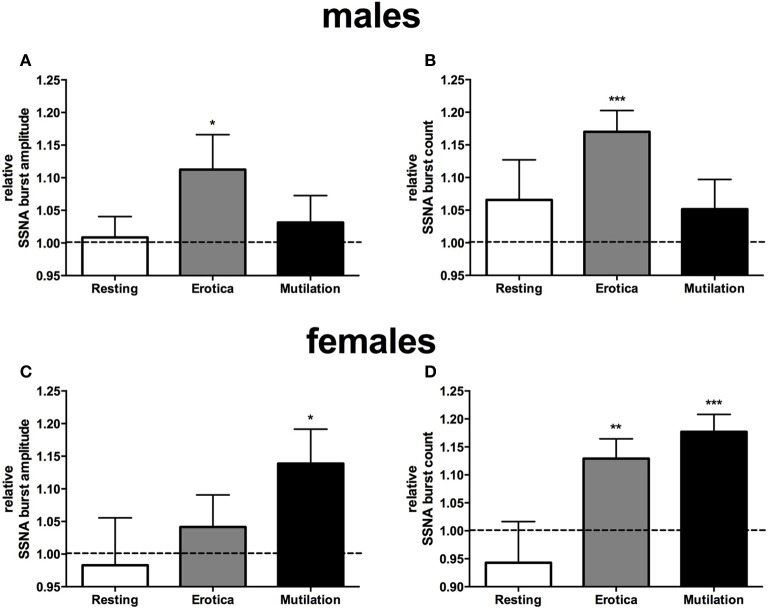
**Mean ± SE changes in burst amplitude (A,C) and frequency (B,D) of skin sympathetic nerve activity, for the resting period, positive images, and negative images, all normalized to the neutral condition, divided into male and female groups**. Erotica had a greater effect on SSNA in males, with burst amplitude and frequency being statistically different from neutral. In females mutilation produced a greater increase in SSNA, with both burst amplitude and frequency being statistically different from neutral. The dashed line represents the neutral value. Statistics refer to differences from neutral. ^*^p < 0.05, ^**^p < 0.01, ^***^p < 0.001.

When subjects were separated into male and females, however, it was clear that there were sex differences in sympathetic reactivity. While blood pressure, heart rate, cutaneous blood flow, and sweat release showed no significant differences between the two groups, SSNA burst amplitude and frequency were significantly different between males and females. For SSNA burst amplitude, compared to the SSNA levels obtained when viewing neutral images the males only showed a significant increase while viewing the positively-charged images (*p* = 0.048), while the females had a significant increase to the negatively-charged images only (*p* = 0.03). For SSNA burst frequency, again the male group only showed a significant increase while viewing the positive images (*p* = 0.0006). However, the female group now showed a significant increase to both the positive (*p* = 0.0064) and negatively-charged images (*p* = 0.0005), although the increase to the mutilation images was greater than that to erotica. Relative changes in SSNA burst count and amplitude, normalized to the neutral condition, are shown for both males and females in Figure 3.

## Discussion

This study has shown that sex differences exist in the sympathetic responses to emotionally-charged visual stimuli, though only when SSNA—measured as total burst count as well as burst amplitude—is measured directly. No significant changes in other physiological parameters, such as blood pressure, heart rate, or respiration were found between the groups. While our previous study was the first to show significant increases in SSNA overall when viewing both positive and negatively-charged images, the current study shows that increases in SSNA were more pronounced in the males when viewing images of erotica, while females had a greater response to images of mutilation. While this study confirms that increases in SSNA can be evoked by visual emotional stimuli (regardless of valence), it indicates that there are sex differences in the response depending on the type of stimulus. Perhaps this is not surprising, but such differences could not be discerned when looking at the indirect markers of sympathetic outflow. Moreover, that there were no significant changes in cutaneous blood flow or sweat release emphasizes the greater sensitivity of direct nerve recordings in the assessment of sympathetic outflow to the skin than indirect measures of cutaneous sympathetic activity.

While a common assumption is held that there are sex differences in emotional development and emotional processing (females are more reactive, perceptive, and expressive with their emotions than males), much of the evidence is provided through self-reported data. It is only recently that, through empirical physiological research, this view appears to have some basis in truth (Kring and Vanderbilt, 1998; Bradley et al., 2001). However, despite this slow emergence that sex differences and the ANS response to emotion is present, there is still no clear evidence of striking sex differences, whether measured through direct or indirect means. Using indirect measurements of sympathetic activation, such as sweat release, during emotional stimuli has yielded some positive and negative findings. Bradley et al. (2001) found that skin conductance responses showed men were more reactive than women to pictures of erotica, with Kring and Vanderbilt (1998) finding that women were more expressive than men, both to positive and negative expressions. However, while Bianchin and Angrilli (2012) found a greater deceleration in heart rate in females for pleasant visual stimuli, no sex differences were found in skin conductance responses. Likewise, Lithari et al. (2010) examined skin conductance responses and event-related EEG potentials (ERP) and found that females responded stronger in terms of ERP amplitudes to unpleasant or high arousing stimuli relative to males, yet found no sex differences in skin conductance responses. This is in agreement with our present study, where we too found that indirect measurements such as sweat release could not differentiate between sexes with either positive or negative images. Moreover, to further confound sex differences and emotion, Vrana and Rollock (2002) studied emotional responses in both white and black (African-American) participants, and found sex differences only in the white participants. Although our study was not designed to address potential racial differences, in the current study, as well as our earlier study (Brown et al., 2012), all of the participants were Caucasian, Mediterranean or Asian; none were Indigenous or African-American.

In recent times, the use of functional neuroimaging has emerged as a technique to assess emotional processing. In particular, investigating sex differences in neural functioning associated with emotional processes has grown, although the findings are not always consistent and study limitations do exist (Wrase et al., 2003; Schienle et al., 2005; McRae et al., 2008; Domes et al., 2010). Nonetheless, there are emerging patterns in sex differences, with females being found to be more emotionally perceptive and experiencing emotions with greater frequency and intensity than males, while males are thought to be more efficient in emotion regulation (Whittle et al., 2011). With reactivity to emotional stimuli, it is widely accepted that males are more responsive to sexually arousing stimuli than females, and this has been reported in both neuroimaging studies as well as physiological studies (Hamann et al., 2004; Allen et al., 2007). However, despite this being widely accepted it is poorly documented, although in the present study sex differences were seen between the positively-charged and negatively-charged images. As a group there were no differences in SSNA responses between positively-charged and negatively-charged images, yet—as noted above—females had a greater response than males to the mutilation images, while males responded more to the erotic images. This suggests that using direct measurements of SSNA, obtained through microneurography, can yield more comprehensive and conclusive results than just using indirect measures, such as heart rate, blood pressure, sweat release and skin blood flow, alone.

## Limitations

While trait variables such as temperament and personality, as well as cultural differences, are always going to be a potential limitation in studies of emotion, the majority of subjects included in the current study consisted of individuals who were not only naïve to the IAPS images but also reported similar reactions to the pictures. When questioned on there reactions at the end of the experiment, all subjects reported being disturbed by the mutilation images, while the majority felt quite neutral toward the erotica images, with no subject being offended by the erotica. Nonetheless, trait differences have the potential to impact on the degree of responses between individuals.

Another limitation of studying the physiological effects of emotionally-charged images is the use of neutral images in between the blocks of emotionally-charged images. While the preceding block of neutral images are used to gauge the extent of responses during the emotionally-charged images, the response to the neutral images in some individuals may be higher than in others depending on the image viewed (i.e., an image of an airplane in an individual who has a fear of flying). As for sex differences, the menstrual cycle and its effect on sympathetic nerve activity as well as emotion is another factor that needs to be taken into account during emotional studies, as differences in physiological functioning during different phases of the menstrual cycle have been found (Goldstein et al., 2005; Carter et al., 2013). For our study however, this was not monitored and the female responses are presented together regardless of the phase of the menstrual cycle; it may well be worth examining the effects of menstrual status in future studies.

## Conclusions

Using intraneural microelectrodes to record directly from postganglionic sympathetic axons directed to the skin, we have shown conclusively that sex differences do exist in the sympathetic neural responses to images of erotica and mutilation. Such differences could not be discerned through the indirect measures of skin sympathetic outflow—sweat release or cutaneous blood flow—as well as other indirect autonomic measures, such as heart rate, blood pressure, and respiration.

### Conflict of interest statement

The authors declare that the research was conducted in the absence of any commercial or financial relationships that could be construed as a potential conflict of interest.
